# ﻿The complete mitochondrial genome of *Siphonariajaponica* (Heterobranchia, Siphonariidae) and its phylogenetic implications

**DOI:** 10.3897/zookeys.1240.141126

**Published:** 2025-06-06

**Authors:** Chao Ma, Lingli Meng, Xiyao Wang, Yimeng Liu, Yaoyao Li, Yunguo Liu

**Affiliations:** 1 College of Life Sciences, Linyi University, Linyi 276005, China Linyi University Linyi China; 2 College of Food Science, Nanchang University, Nanchang 330047, China Nanchang University Nanchang China

**Keywords:** Heterobranchia, mitogenome, molecular phylogeny, next-generation sequencing, *Siphonariajaponica*, structural feature

## Abstract

The gastropod *Siphonariajaponica*, belonging to the family Siphonariidae, is an important grazer in the rocky intertidal zone of China, possessing significant ecological functions and medicinal value. To enrich the diversity of mitochondrial genomes in Gastropoda and to gain insights into their phylogenetic relationships, the mitochondrial genome of *S.japonica* (13,966 bp) was sequenced using next-generation sequencing. The genome comprises 13 protein-coding genes, 22 tRNA genes, and 2 rRNA genes; the gene organization, nucleotide composition, and codon usage are consistent with other Heterobranchia species. Notably, comparisons of mitochondrial gene rearrangements indicated that Siphonariidae exhibit extensive and irregular rearrangements within Heterobranchia. Further, we reconstructed the most comprehensive phylogenetic analysis of Gastropoda based on 13 concatenated protein-coding genes. Each subclass of Gastropoda formed a monophyletic clade, with Heterobranchia positioned at the base of the tree, and *S.japonica* has a sister relationship with *S.pectinata*. The findings of this study will contribute to a better understanding of the characteristics of the *S.japonica* mitogenome, provide valuable insights into the phylogenetic relationships within Gastropoda, and underscore the utility of mitochondrial genomes in systematics.

## ﻿Introduction

The mitochondrial genome (mt) of metazoans is generally characterized as a small, closed-circular structure, typically ranging from 15 to 20 kb in size. It encodes 13 protein-coding genes (PCGs), 22 transfer RNAs (tRNAs), two ribosomal RNAs (rRNAs), and a non-coding region (NCR) (Boore 1999). Mitogenomes have been extensively employed to elucidate phylogenetic relationships and identify common species due to its simple structure, abundant copies, rapid evolutionary rate, and ease of isolation (Wang et al. 2014; Minton et al. 2016; Yeung et al. 2020; Kosicka et al. 2022; Zhao et al. 2023; Xu et al. 2024). The advent of novel sequencing technologies and the substantial reduction in costs associated with next-generation sequencing have led to a rapid increase in the number of available mitogenomes. White et al. (2011) analyzed the complete mitogenomes of ten pulmonate species to explore their phylogenetic relationships. Nevertheless, due to low support values and some incongruence between analyses based on complete mitochondrial genomes and individual genes, some deeper nodes remain uncertain. Thus, more complete mitochondrial genomes and markers of pulmonates and related species are required to address the deep node issues.

In recent years, the determination of mitochondrial genome and phylogenetic studies have progressed rapidly, and a new classification divides the gastropods into six subclasses, namely Vetigastropoda, Neomphaliones, Caenogastropoda, Neritimorpha, Patellogastropoda and Heterobranchia (Bouchet et al. 2017). *Siphonariajaponica* (Donovan, 1824), belonging to the Siphonariidae of Heterobranchia, is a common marine gastropod species widely distributed in the middle and upper tidal zones of rocky intertidal areas in China and throughout Southeast Asia (Cai et al. 2013; Wang et al. 2015; Dong et al. 2016). As an important grazer in the rocky intertidal zone of China, *S.japonica* plays a crucial regulatory role in the composition and abundance of intertidal algae, serving an important ecological function (Chan 2003). Additionally, *S.japonica* also holds significant application value, in that the polypropionate compounds isolated from its body are known for clearing inflammation and detoxification, and have considerable medicinal value (Zeng et al. 1992). In recent years, research on *S.japonica* mainly encompasses aspects such as nutritional components (Zeng et al. 1992), population ecology (Chen et al. 2017; Wang et al. 2020), genetic divergence (Yokogawa et al. 2010), and the influence of environmental changes on the growth and development of larvae (Wang et al. 2017; Wang 2019). Nevertheless, few have conducted in-depth studies on the taxonomy and systematics of the family Siphonariidae.

Due to the fact that the shells of shellfish are prone to the influence of environmental factors, the color and pattern of shells frequently undergo changes, with an increasing number of intraspecific variations (Miao et al. 2023). Additionally, many species exhibit homonymy or synonymy. Consequently, classification merely relying on the appearance of shells often gives rise to errors and confusion (Ossenbrügger et al. 2023). Yokogawa et al. (2010) employed allozymes as genetic markers to conduct genetic analyses on two forms of *S.japonica*. They discovered that the intraspecific genetic differences between them were considerable and opined that the tall-shell and short-shell forms of *S.japonica* should be regarded as distinct species, demanding a taxonomic revision. Therefore, the combination of morphological identification and molecular biological approaches can enhance the accuracy of species delimitation. For example, Zheng et al. (2021) utilized the DNA barcode mitochondrial *COI* gene to identify common limpet species on the bedrock coast of Lianyungang and identified one *S.japonica* and two *Patella* species. Nakayama et al. (2017) employed DNA barcodes to identify the cryptic limpet species *Lottiakogamogai* (Sasaki & Okutani, 1993). Ossenbrügger et al. (2023) investigated the species diversity of *Siphonaria* in Seychelles Bank, Indian Ocean, and beyond using *16S* rRNA gene; they found their specimens formed three distinct clades, whereas comparing morphological and molecular data implied there might be more than three species. Regardless of those previous studies, the current species delimitation of *S.japonica* still relies on a single gene (Zheng et al. 2021), and there has been no report regarding its mitochondrial genome thus far.

In this study, we applied next-generation sequencing to obtain the complete mitogenome of *S.japonica*, analyzed its respective characteristics, and reconstructed the molecular phylogenetic relationships of Gastropoda. The molecular data presented in this study will contribute to a better understanding of the characteristics of the gastropod mitogenomes. The goal of our study was to place the new mitogenome of *S.japonica* within the context of the known mitogenomes of Gastropoda by performing mitogenomic and phylogenetic analyses. The findings will provide valuable insights into the evolution and phylogeny of gastropods and contribute to the development of germplasm resources.

## ﻿Material and methods

### ﻿Sampling and DNA extraction

In April 2023, five adult specimens of *S.japonica* were collected from Weifang City, Shandong Province, China (37°11'29"N, 119°11'57"E). Species identification was conducted following descriptions and illustrations provided in Zheng et al. (2021) and the World Register of Marine Species (WoRMS; https://www.marinespecies.org). Muscle samples were deposited in 95% ethanol for subsequent DNA extraction. Genomic DNA was isolated using TIANamp Genomic DNA Kit (Tiangen, Beijing, China) according to the instructions. DNA sample quantity and quality were characterized by the Nano-Drop 2000 spectrometer (Thermo Scientific, Waltham, MA, USA) and 1% gel electrophoresis.

### ﻿DNA sequencing and genome assembly

The complete mitogenome sequence of *S.japonica* was obtained through next-generation sequencing. The total DNA was sheared to 300–500 bp fragments via ultrasound, and the VAHTS Universal Plus DNA Library Prep Kit for Illumina was used for library construction. The procedures involved: end-repair of DNA fragments, addition of A at the 3’ end, ligation of sequencing adapters, and recovery of the target fragments by agarose gel electrophoresis. The target fragments were then amplified by PCR, and ultimately, the sequencing library was constructed. Before sequencing, the library underwent quality control checks. The library was sequenced as 150 bp paired-end runs on Illumina Novaseq 6000 platform. Finally, 78,191,908 raw reads were generated. After removing the adapters and low-quality regions using FastQC (Andrews 2018) and Cutadapt (Martin 2011) the clean reads underwent de novo assembly using NOVOPlasty software (Nicolas et al. 2017).

### ﻿Mitogenome annotation and structural analysis

Annotation of the mt genome of *S.japonica* was performed using Mitos2 (http://mitos2.bioinf.uni-leipzig.de/index.py) (Bernt et al. 2013). The positions of protein-coding genes (PCGs), transfer (t)RNAs, and ribosomal (r)RNAs genes were further confirmed manually using BioEdit program (Hall 1999) and BLAST to search for homologous sequences. Transfer RNA genes were identified and their secondary structures were predicted using RNAfold web server (http://rna.tbi.univie.ac.at/cgi-bin/RNAWebSuite/RNAfold.cgi) (Lorenz et al. 2011). The relative synonymous codon usage (RSCU) of PCGs, along with the nucleotide composition of the mitochondrial genomes, PCGs, rRNA, and tRNA genes were calculated using MEGA X (Kumar et al. 2018). Strand asymmetry was described using the following formulas: AT-skew = (A - T) / (A + T) and GC-skew = (G - C) / (G + C) (Hassanin et al. 2005). The circular genome map of *S.japonica* was generated using the online server Proksee (https://proksee.ca/) (Grant et al. 2023). The fully annotated mitogenome was then submitted to GenBank using the Sequin tool (http://www.ncbi.nlm.nih.gov/Sequin/) to obtain the GenBank accession number.

### ﻿Molecular phylogenetic analyses

To determine the phylogenetic position of *S.japonica*, we performed phylogenetic analyses using PCGs of Gastropoda. This included the newly sequenced *S.japonica* as well as genome sequences from 140 gastropod species obtained from GenBank, as well as the bivalve species, *Venustaconchaellipsiformis* (Conrad, 1836) (NC_013659), which was used as an outgroup (Suppl. material 1). Mitochondrial genomic data were processed using PhyloSuite (Zhang et al. 2020). Firstly, the 13 PCGs were aligned each differently using MAFFT v. 7 (Katoh and Standley 2013) with default parameters, then the alignment results were optimized using MACSE v. 2 (Ranwez et al. 2018). Ambiguously aligned positions were removed using Gblock v. 0.91 (Talavera and Castresana 2007). Subsequently, the PCGs of all species were concatenated into a large dataset. Phylogenetic analyses were conducted using both maximum likelihood (ML) and Bayesian inference (BI) methods. Codon positions for each PCG (13 genes × 3 codons = 39 partitions) were predefined. The best-fit models and partitioning schemes were determined using PartitionFinder v. 2.1.1 (Lanfear et al. 2017) via the Bayesian information criterion (BIC) (Suppl. material 2). ML analysis was carried out using IQ-TREE v. 2.0 (Nguyen et al. 2015) with 1000 bootstrap replicates based on partitioned nucleotide alignments. BI analysis was conducted with 10,000,000 generations using MrBayes v. 3.2.6 (Ronquist et al. 2012), employing four simultaneous Markov chains with sampling every 1000 generations, and discarding the first 25% generations as burn-in. The BI tree was considered reliable, as the standard deviation of split frequencies was below 0.01. The resulting trees were visualized in iTOL v. 6 (Ivica and Peer 2024).

## ﻿Results and discussion

### ﻿Genome organization and composition

**Figure 1.**
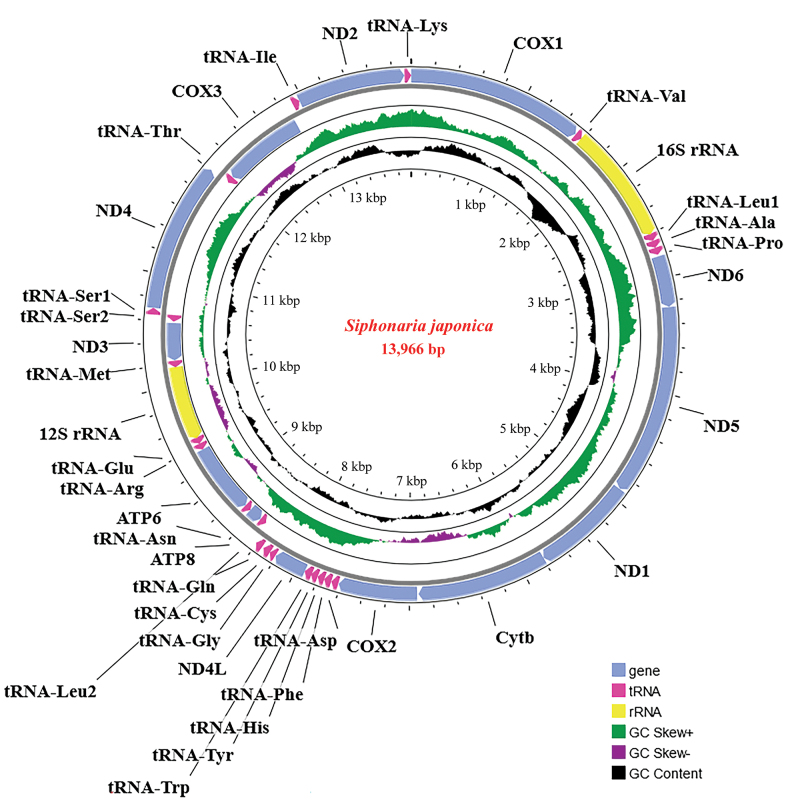
The organization of the mitogenome of *Siphonariajaponica*.

The complete mitochondrial genome of *S.japonica* (PQ249683) was 13,966 bp in length, consisting of 13 PCGs, 22 transfer RNA genes and 2 ribosomal RNA genes (12S and 16S) (Fig. 1). The 25 genes (9 PCGs, one rRNAs, and 15 tRNAs) were coded with the heavy (H-) strand, while the remaining (4 PCGs, one rRNA, and 7 tRNAs) were encoded with the light (L-) strand (Fig. 1, Table 1). The gene arrangement on both the L and H strands was canonical and consistent with that of other Heterobranchia gastropods. The mt genome of *S.japonica* exhibits a total of 39 bp of overlap sequences (Table 1), which were identified at ten gene junctions, ranging from 1 bp to 10 bp, the longest overlap was located between tRNA^Ser1^ and tRNA^Ser2^. In addition, 16 intergenic spacers were observed, totaling 170 bp (Table 1), with individual spacer lengths varying from 1 bp to 53 bp. The longest intergenic spacer was located between *COX3* and tRNA^Ile^.

**Table 1.** List of annotated mitochondrial genes and their key features in *S.japonica*.

Gene	Position		Length	Start codon	Stop codon	Anticodon	Intergenic nucleotides^a^	Strand^b^
	Start	End						
COX1	1	1525	1525	TTG	T--		0	H
tRNA^Val^	1529	1589	61			TAC	3	H
16S rRNA	1588	2610	1023				-2	H
tRNA^Leu1^	2605	2668	64			TAG	-6	H
tRNA^Ala^	2669	2731	63			TGC	0	H
tRNA^Pro^	2732	2794	63			TGG	0	H
ND6	2810	3253	444	ATG	TA-		15	H
ND5	3255	4907	1653	ATG	TAG		1	H
ND1	4909	5794	886	ATG	T--		1	H
Cytb	5795	6922	1128	TTG	TAA		0	H
COX2	6935	7615	681	ATG	TAA		12	H
tRNA^Asp^	7620	7681	62			GTC	4	H
tRNA^Phe^	7683	7746	64			GAA	1	H
tRNA^His^	7745	7805	61			GTG	-2	H
tRNA^Tyr^	7806	7866	61			GTA	0	H
tRNA^Trp^	7861	7925	65			TCA	-6	H
ND4L	7925	8207	283	ATG	T--		-1	H
tRNA^Gly^	8212	8265	54			TCC	4	H
tRNA^Cys^	8266	8331	66			GCA	0	H
tRNA^Gln^	8348	8409	62			TTG	16	H
tRNA^Leu2^	8446	8507	62			TAA	36	L
ATP8	8508	8635	128	ATG	T--		0	L
tRNA^Asn^	8636	8697	62			GTT	0	L
ATP6	8698	9358	661	ATG	T--		0	L
tRNA^Arg^	9359	9420	62			TCG	0	L
tRNA^Glu^	9420	9484	65			TTC	-1	L
12S rRNA	9477	10172	696				-8	L
tRNA^Met^	10171	10232	62			CAT	-2	L
ND3	10234	10584	351	ATG	TA-		1	L
tRNA^Ser2^	10598	10659	62			TGA	13	L
tRNA^Ser1^	10650	10707	58			GCT	-10	H
ND4	10707	12008	1302	TTG	TAG		-1	H
tRNA^Thr^	12016	12079	64			TGT	7	L
COX3	12081	12858	778	ATG	TAA		1	L
tRNA^Ile^	12912	12974	63			GAT	53	H
ND2	12975	13907	933	ATG	TAG		0	H
tRNA^Lys^	13910	13966	57			TTT	2	H

^a^ Negative value signifies overlapping sequences between adjacent genes. ^b^ “H” and “L” denote the heavy and light strands, respectively.

The nucleotide composition of the mitochondrial genome consists of 28.66% A, 36.86% T, 15.95% C, and 18.53% G. The A + T content (65.52%) is significantly higher than the GC content (34.48%), suggesting the presence of strand asymmetry or strand-specific nucleotide bias. The A+T content was 65.41% in the PCGs, 66.67% in rRNAs, and 63.41% in the tRNAs. Similarly, a comparison among the mitochondrial genomes of 26 Heterobranchia species showed essentially the same nucleotide composition (Suppl. material 3). The AT and GC-skew of *S.japonica* was -0.125 and -0.075, respectively, showing a skew from A towards T but a minor skew from C towards G. This strand asymmetry has also been documented in other gastropod taxa (Williams et al. 2014; Gaitán-Espitia et al. 2019). Among representative Heterobranchia species, the AT-skew ranged from -0.210 in *S.gigas* (G. B. Sowerby I, 1825) to 0.137 in *Nembrothakubaryana* (Bergh, 1877), while the GC-skew varied from -0.127 in *N.kubaryana* to 0.215 in *S.gigas* (Suppl. material 3).

### ﻿Protein-coding genes

**Figure 2.**
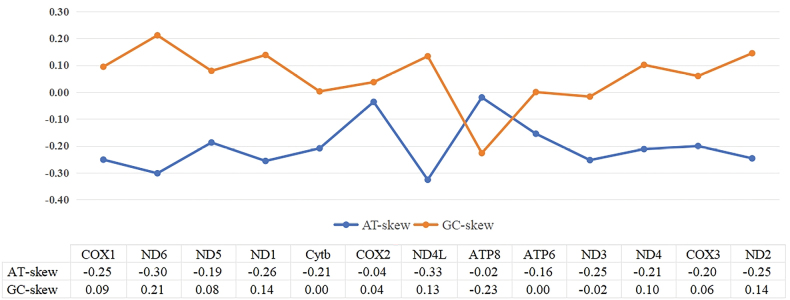
Graphical representation of AT and GC-skew in all 13 PCGs in the mitochondrial genome of *S.japonica*.

The total length of all PCGs was 10752 bp and accounted for 76.99% of the whole mitogenome. The coding regions ranged in size from 128 bp (*ATP8*) to 1653 bp (*ND5*). This pattern aligns with other Heterobranchia gastropods, in which *ATP8* is typically the shortest and *ND5* the longest of the PCGs (White et al. 2011). The total A+T content of 13 PCGs in *S.japonica* was 65.41%, ranging from 63.08% (*COX1*) to 68.20% (*ND4L*). The GC skews in these PCGs of *S.japonica* were wavering near zero, with the majority of the genes displaying positive skew, except for the *ND3* and *ATP8* regions. The AT skews were all negative (Fig. 2). This situation was consistent with most gastropods (Yang et al. 2018; Feng et al. 2020).

Similar to other Heterobranchia mitogenomes, the majority of PCGs started with ATG as initiation codons, except for *COX1*, *Cytb* and *ND4* starting with TTG. It is quite common for these genes to use unconventional start codons in gastropod mitogenomes (Feng et al. 2020; Miao et al. 2023). As for the termination codons, PCGs employ the canonical termination codon (TAA and TAG) and incomplete termination codons (TA and T) (Table 1). Incomplete termination codons, such as TA or T, are typically extended to TAA via post-transcriptional polyadenylation, and it is commonly recognized in the mitochondrial genomes of gastropods (Suppl. material 4) (Feng 2021).

**Figure 3.**
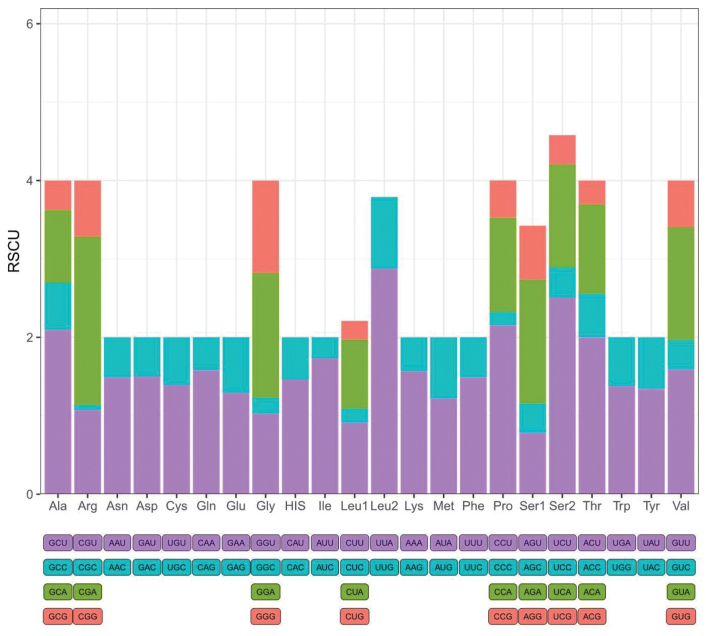
The relative synonymous codon usage (RSCU) of the mitochondrial PCGs of *S.japonica*.

Codon usage of PCGs in *S.japonica* and other gastropods is presented in Fig. 3 and Suppl. material 5. The mt genomes of *S.japonica* encode a total of 3584 amino acids, among which the most frequently used were serine (13.25%), leucine (12.47%), phenylalanine (8.81%), and isoleucine (6.06%), while glutamine was rare (1.40%). The distribution of amino acids and their relative frequencies in *S.japonica* was essentially consistent with that of other Siphonariidae species (White et al. 2011). Furthermore, codon usage exhibits a bias towards A and T at the third position of the most commonly used codons (UUA-Leu, CCU-Pro, UCU-Ser, CGA-Arg, ACU-Thr), a trend consistent with previous findings in Heterobranchia gastropods (Suppl. material 5) (White et al. 2011).

### ﻿Transfer and ribosomal RNA genes

**Figure 4.**
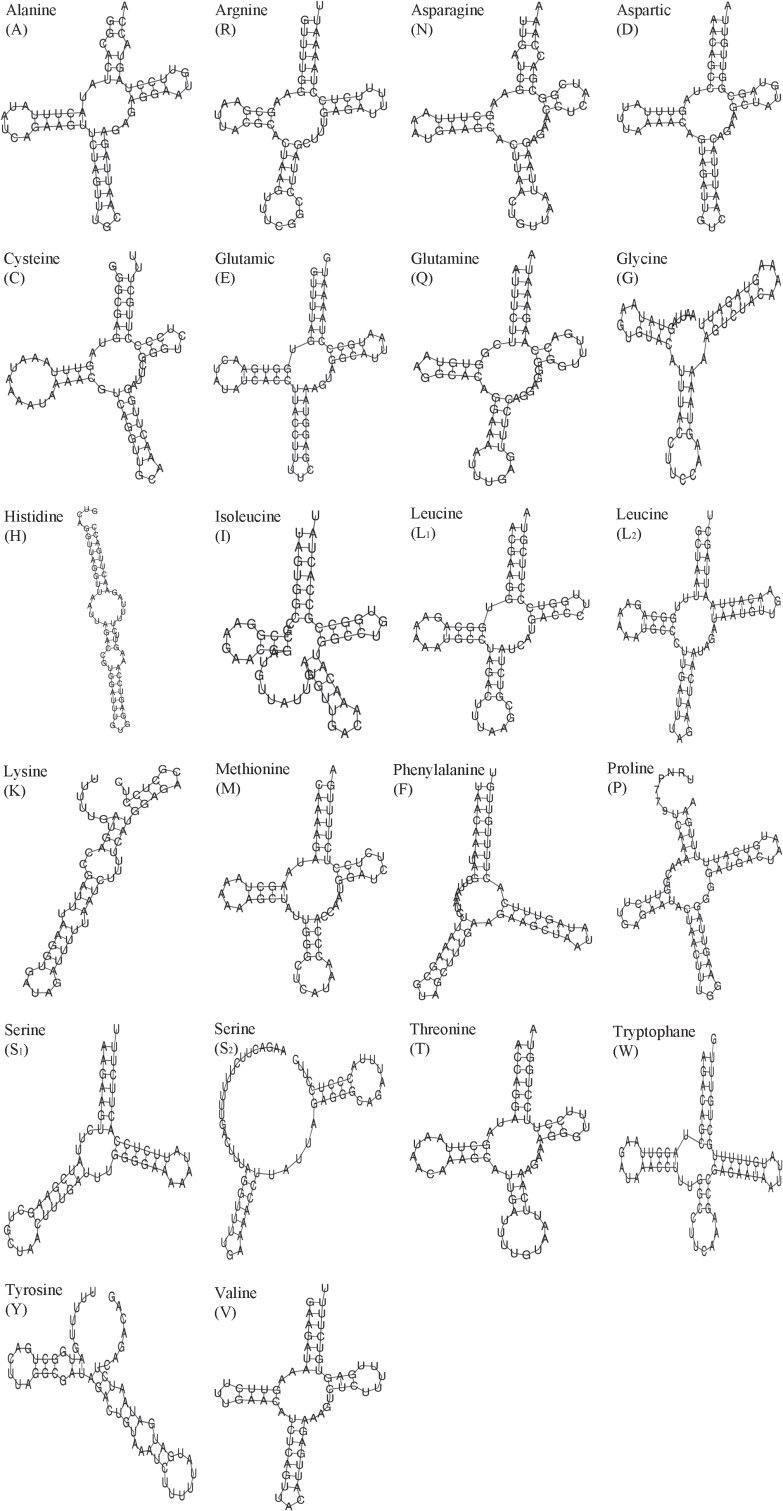
Putative secondary structures for 22 tRNA genes in the mitogenome of *S.japonica*.

*Siphonariajaponica* contained 22 tRNAs (1361 bp), accounting for 9.7% of the mt genome. The average length ranged from 54 bp (tRNA^Gly^) to 66 bp (tRNA^Cys^) (Table 1). Fifteen of the tRNA genes were encoded by the H chain, and the remaining seven were located on the L chain (Table 1). Leucine was encoded by two anticodons (UAG, UAA) and Serine was encoded by UGA and GCU. The presence of multiple tRNAs recognizing different anticodons is a common feature of gastropod mitogenomes (Xie 2019; Feng et al. 2020). Most tRNA genes could be folded into canonical cloverleaf secondary structures except tRNA^Gly^, tRNA^His^, tRNA^Ile^, tRNA^Lys^, tRNA^Phe^, tRNA^Ser^ and tRNA^Tyr^ (Fig. 4). To possess functions similar to normal tRNAs, these aberrant tRNAs might require co-evolved interaction factors or post-transcriptional RNA editing (Masta and Boore 2004; Sun et al. 2016).

Consistent with other gastropods, two rRNA genes were present in *S.japonica*. The large ribosomal RNA gene (*16S* rRNA), measuring 1023 bp in length (1588~2610 bp), is located between tRNA^Val^ and tRNA^Leu^ (Table 1); the small (*12S* rRNA), with a length of 696 bp (9477~10172 bp), is located between tRNA^Glu^ and tRNA^Met^ (Table 1). The AT content of the two rRNA genes was 66.67%, which is higher than that of the entire mitogenome at 65.52%. The AT-skew and GC-skew of rRNAs were 0.025 and 0.140, respectively.

### ﻿Gene rearrangement of Heterobranchia

Gastropods, as a major dominant group among invertebrates, have a gene structure similar to that of other invertebrates. Among them, the number of tRNA genes often exhibits variability (Hu and Wang 2019; Kumar et al. 2020; Yang et al. 2020). The mitogenome of gastropods features a compact structure, small gene intervals, significant variations in the control region, and numerous gene overlaps, where many species present these gene rearrangement phenomena (Feng et al. 2020; Machkour-M’Rabet et al. 2021).

**Figure 5.**
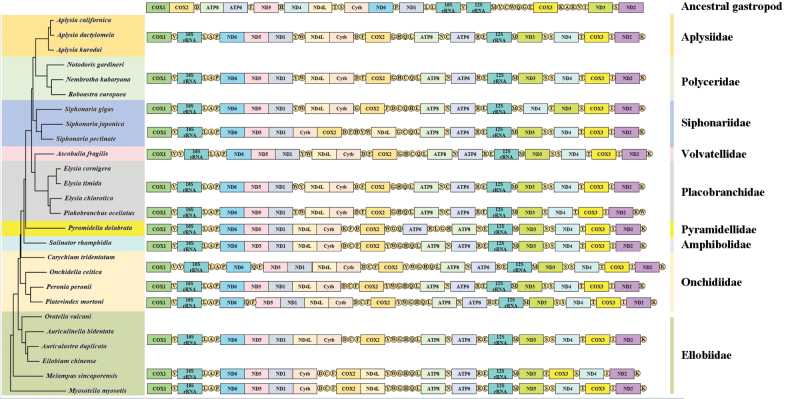
Linear sequencing map of mitogenomes of Heterobranchia.

We took the *COX1* gene as the starting point and compared the gene order of currently known sequences of Heterobranchia with that of the ancestral sequence of gastropods (Fig. 5) (Kumar et al. 2020). Compared with the ancestral sequence, the genomic orders of Heterobranchia have all undergone significant alterations. Among the nine families under Heterobranchia in this study (Fig. 5), the sequences of Aplysiidae and Polyceridae are relatively conserved, the gene orders of all species within these families are identical, and other families all display varying degrees of rearrangement. The Siphonariidae family has undergone a high degree of rearrangement. The gene orders of *S.japonica* that we measured are the same as *S.pectinata*, but they are significantly different from those of *S.gigas*. In the PCGs, *Cytb*, *COX2*, and *ND4L* have undergone obvious displacements, and rearrangement is also manifested in the positions of tRNAs. This high degree of rearrangement in the family Siphonariidae has also occurred in the family Lottiidae (Miao et al. 2023); we speculate that perhaps the increase in the evolutionary rate has led to the high degree of rearrangement of the mitogenome of Siphonariidae.

Previous studies on the mitogenomes of Gastropoda have demonstrated that almost all subclasses possess unique gene order patterns, while lineages within each subclass are relatively conserved (White et al. 2011; Osca et al. 2015; Uribe et al. 2016). Within these major lineages, gene rearrangements mostly involve the translocation of some tRNAs and protein-coding genes or the inversion of gene clusters, and gene rearrangements mostly occur between higher taxa. However, a unique mitochondrial gene order pattern of *Physellaacuta* (Draparnaud, 1805) belonging to Heterobranchia is significantly different from that of its closely related species (Nolan et al. 2014), and Feng et al. (2020) studied gene arrangements of Patellogastropoda and found a highly irregular rearrangement of mitochondrial genes in Lottiidae. These findings reveal that some lineages may have unique evolutionary patterns, resulting in the rearrangement of their unique mitochondrial genes.

### ﻿Phylogenetic analysis of Gastropoda

**Figure 6.**
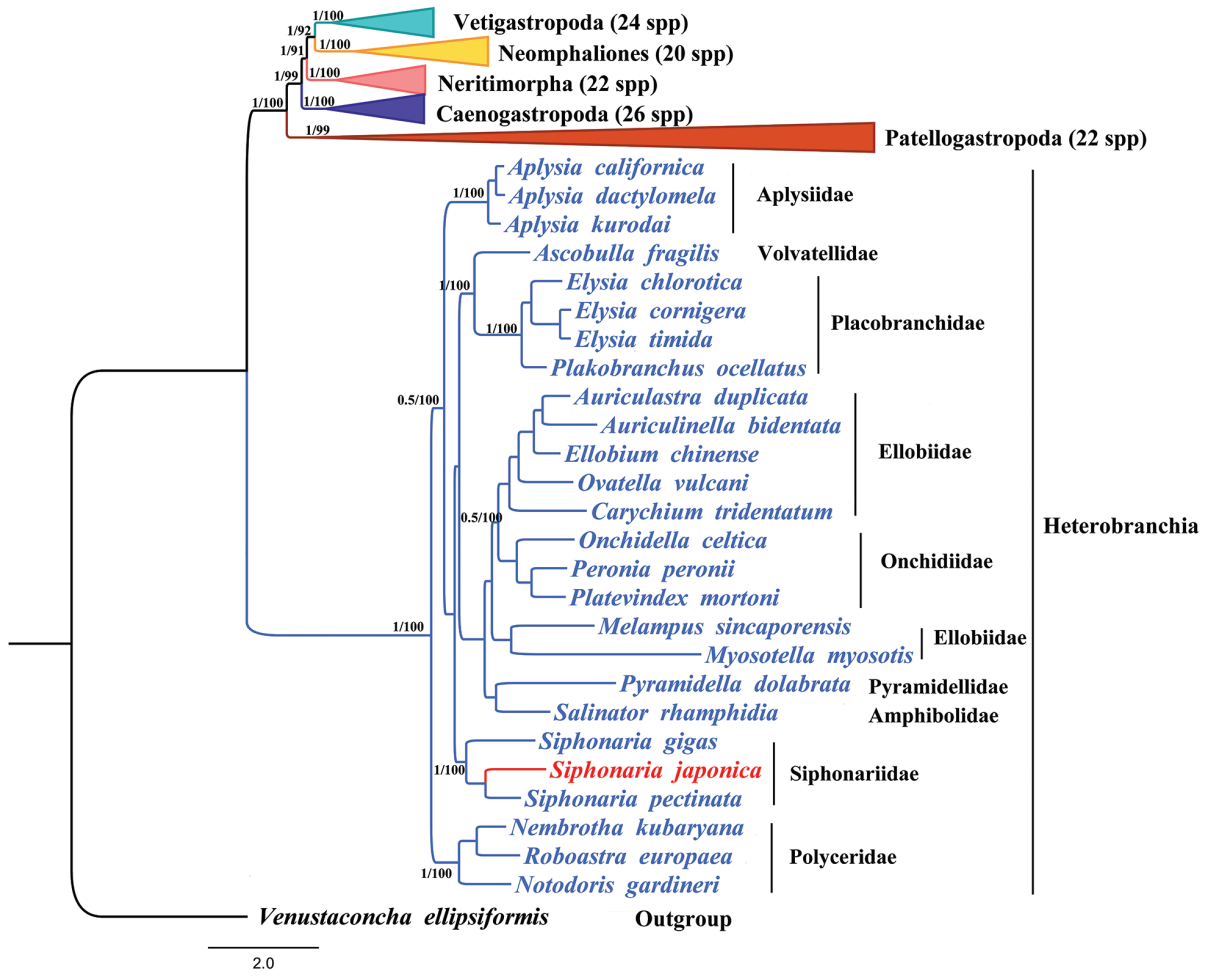
Phylogenetic tree inferred from 13 PCGs of the mitochondrial genome using BI and ML analysis. Numbers along branches represent bootstrap support values of the BI/ML tree.

The systematic relationships within Gastropoda are a prominent topic in molecular phylogenetic research (Nakano and Sasaki 2011; Yang et al. 2018; Uribe et al. 2019; Feng et al. 2020). We used BI and ML methods to reconstruct a phylogenetic tree using 13 protein-coding gene sequences from 140 species within Gastropoda. The topologies generated by both methods were largely congruent (Fig. 6, Suppl. materials 6, 7), with the majority of clades receiving strong support (Bayesian posterior probability (BPP) = 1, Bootstrap (BS) = 100).

The results of maximum likelihood and Bayesian analysis showed a stable evolutionary tree topology of Gastropoda, featuring six major branches corresponding to six subclasses, each forming a monophyletic clade (Fig. 6). The higher-level phylogeny showed the following clade structure: (((((Vetigastropoda + Neomphaliones) + Neritimorpha) + Caenogastropoda) + Patellogastropoda) + Heterobranchia), and Vetigastropoda and Neomphaliones being sister taxa to each other. This topology is consistent with the topology of the Gastropoda phylogenetic tree constructed by Feng et al. (2021). Arquez et al. (2014) analyzed the evolution of five gastropod subclasses using mitochondrial genomes, revealing that Neritimorpha and Vetigastropoda form a sister group, which aligns with our findings. In contrast, Osca et al. (2014) identified Caenogastropoda and Neritimorpha as sister groups, with Vetigastropoda following. Uribe et al. (2016) introduced Neomphalina as a subclass, reaching similar conclusions to Osca et al. (2014). Sun et al. (2019) reclassified Cocculiniformia and Neomphalina into a new subclass, Neomphalines, whose evolutionary relationships matched those in our study. Our branches also share similarities with those described by Feng et al. (2020), with slight discrepancies regarding Patellogastropoda and Heterobranchia. Miao et al. (2023) positioned Patellogastropoda as the outermost subclass, but their BI analysis was affected by long-branch attraction (LBA), leading to incorrect inter-subclass relationships. Feng et al. (2022) observed that Patellogastropoda divided into two branches on opposite sides of Heterobranchia, while in our study, Patellogastropoda formed a monophyletic group not separated by Heterobranchia. As noted by Uribe et al. (2019) and Nakano and Sasaki (2011), variations in phylogenetic analysis outcomes are often due to differences in gene selection, taxon sampling, and the phylogenetic information contained within the genes. These factors underscore the need for cautious interpretation of gastropod phylogenies.

In both ML and BI analyses, within Heterobranchia, Ellobiidae and Onchidiidae are sister groups, with Polyceridae occupying a basal position, showing similarities to those reported by Feng et al. (2021). In Siphonariidae, *S.pectinata* and *S.japonica* sequenced in this study constitute sister taxa. This study reinforces the significance of Heterobranchia species in understanding the evolutionary development of gastropods. Future mitochondrial genome sequencing efforts will be necessary to broaden the species sample and deepen our understanding of gastropod phylogeny and evolution.

## ﻿Conclusion

This study presents the complete mitochondrial genome of *S.japonica*, which is 13,966 bp in length. Its gene organization, nucleotide composition, tRNA secondary structure, and codon usage were analogous to those of other Heterobranchia mitogenomes. Species within the family Siphonariidae demonstrated a relatively broad range of irregular rearrangements in Heterobranchia. Phylogenetic analysis confirmed the phylogenetic position of *S.japonica* within Heterobranchia, with the results largely agreeing with previous molecular phylogenetic relationships within Gastropoda. Nevertheless, due to the scarcity of sufficient mitochondrial genomes, the basal subclass of Gastropoda is still controversial. Generating comprehensive molecular data for Gastropoda species is indispensable. The outcomes of this study supplement the mitogenome database of the family Siphonariidae, and will better facilitate our molecular identification, provide more assistance for further investigations on the molecular taxonomy and genetic diversity of Gastropoda, and provide critical insights for the development of more effective conservation strategies.

## ﻿Additional information

### Author ORCIDs

Chao Ma https://orcid.org/0000-0001-7181-5109

### Data availability

All of the data that support the findings of this study are available in the main text or Supplementary Information.

## Supplementary materials

Supplementary material 1 List of species used in the phylogenetic analyses Data type xlsx https://binary.pensoft.net/file/1346634This dataset is made available under the Open Database License (http://opendatacommons.org/licenses/odbl/1.0/). The Open Database License (ODbL) is a license agreement intended to allow users to freely share, modify, and use this Dataset while maintaining this same freedom for others, provided that the original source and author(s) are credited.Chao Ma, Lingli Meng, Xiyao Wang, Yimeng Liu, Yaoyao Li, Yunguo Liu

Supplementary material 2 The best-fit nucleotide substitution models and partitioning schemes determined with PartitionFinder v. 2.1.1. Data type xlsx https://binary.pensoft.net/file/1346635This dataset is made available under the Open Database License (http://opendatacommons.org/licenses/odbl/1.0/). The Open Database License (ODbL) is a license agreement intended to allow users to freely share, modify, and use this Dataset while maintaining this same freedom for others, provided that the original source and author(s) are credited.Chao Ma, Lingli Meng, Xiyao Wang, Yimeng Liu, Yaoyao Li, Yunguo Liu

Supplementary material 3 Nucleotide composition of the mitochondrial genome in different Heterobranchia species Data type xlsx https://binary.pensoft.net/file/1346636This dataset is made available under the Open Database License (http://opendatacommons.org/licenses/odbl/1.0/). The Open Database License (ODbL) is a license agreement intended to allow users to freely share, modify, and use this Dataset while maintaining this same freedom for others, provided that the original source and author(s) are credited.Chao Ma, Lingli Meng, Xiyao Wang, Yimeng Liu, Yaoyao Li, Yunguo Liu

Supplementary material 4 Comparison of start and stop codons of PCGs of 26 Heterobranchia species Data type xlsx https://binary.pensoft.net/file/1346637This dataset is made available under the Open Database License (http://opendatacommons.org/licenses/odbl/1.0/). The Open Database License (ODbL) is a license agreement intended to allow users to freely share, modify, and use this Dataset while maintaining this same freedom for others, provided that the original source and author(s) are credited.Chao Ma, Lingli Meng, Xiyao Wang, Yimeng Liu, Yaoyao Li, Yunguo Liu

Supplementary material 5 The RSCU value of the complete PCGs of Siphonariidae species Data type xlsx https://binary.pensoft.net/file/1346638This dataset is made available under the Open Database License (http://opendatacommons.org/licenses/odbl/1.0/). The Open Database License (ODbL) is a license agreement intended to allow users to freely share, modify, and use this Dataset while maintaining this same freedom for others, provided that the original source and author(s) are credited.Chao Ma, Lingli Meng, Xiyao Wang, Yimeng Liu, Yaoyao Li, Yunguo Liu

Supplementary material 6 Phylogenetic tree inferred from nucleotide sequences of 13 PCGs of the mitogenome using BI analysis Data type jpg Explanation note The numbers along branches indicate posterior probability values. https://binary.pensoft.net/file/1346639This dataset is made available under the Open Database License (http://opendatacommons.org/licenses/odbl/1.0/). The Open Database License (ODbL) is a license agreement intended to allow users to freely share, modify, and use this Dataset while maintaining this same freedom for others, provided that the original source and author(s) are credited.Chao Ma, Lingli Meng, Xiyao Wang, Yimeng Liu, Yaoyao Li, Yunguo Liu

Supplementary material 7 Phylogenetic tree inferred from nucleotide sequences of 13 PCGs of the mitogenome using ML analysis Data type jpg Explanation note The numbers along branches indicate bootstrap support values. https://binary.pensoft.net/file/1346640This dataset is made available under the Open Database License (http://opendatacommons.org/licenses/odbl/1.0/). The Open Database License (ODbL) is a license agreement intended to allow users to freely share, modify, and use this Dataset while maintaining this same freedom for others, provided that the original source and author(s) are credited.Chao Ma, Lingli Meng, Xiyao Wang, Yimeng Liu, Yaoyao Li, Yunguo Liu
