# Supplementary figures and images for: ﻿The complete mitochondrial genome of Siphonariajaponica (Heterobranchia, Siphonariidae) and its phylogenetic implications
Source: Zookeys. 2025 Jun 6;1240:257–76. doi: 10.3897/zookeys.1240.141126 (PMC12166384; doi:10.3897/zookeys.1240.141126)

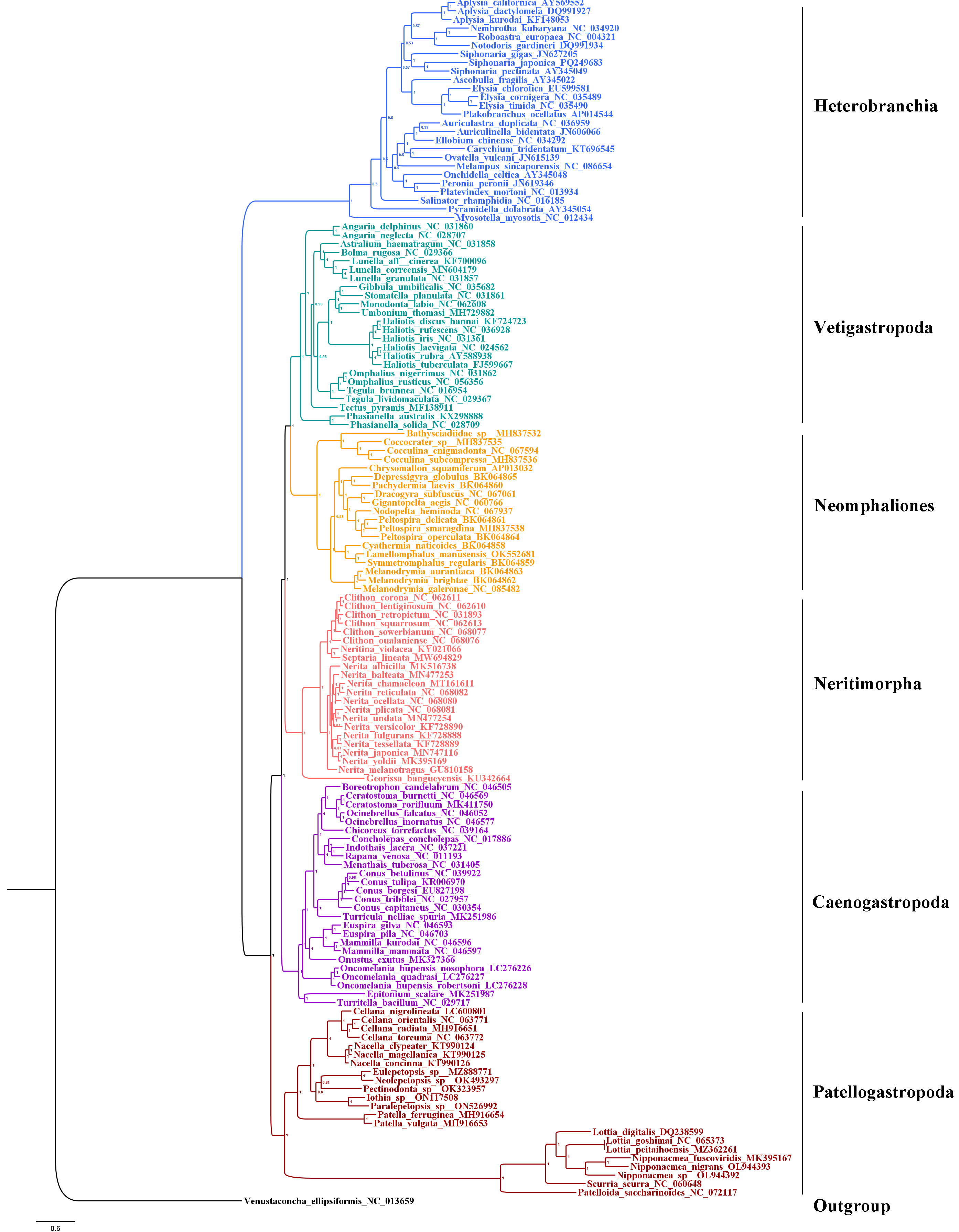

Supplement: Supplementary material 6 — Phylogenetic tree inferred from nucleotide sequences of 13 PCGs of the mitogenome using BI analysis [file zookeys-1240-257_article-141126__-s006.jpg]

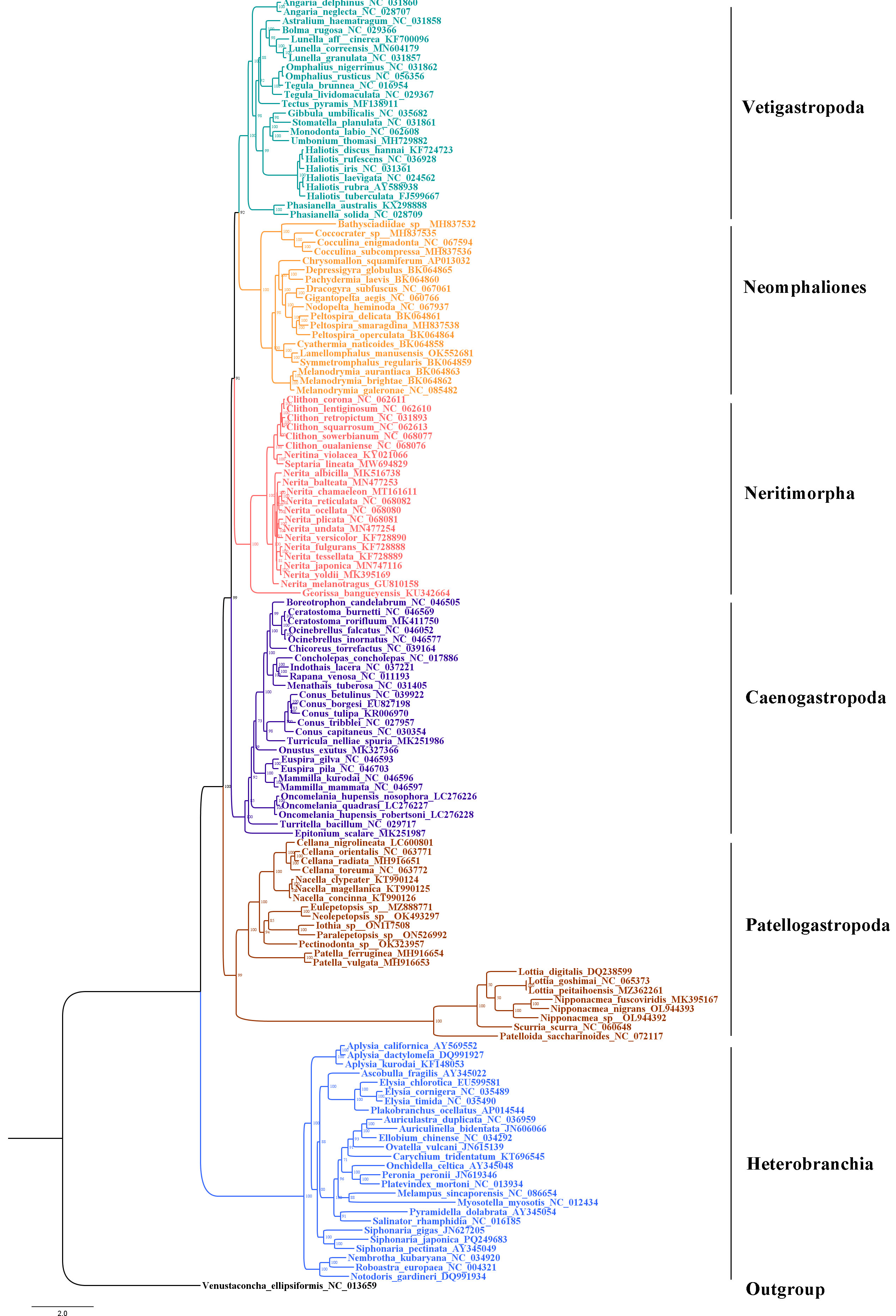

Supplement: Supplementary material 7 — Phylogenetic tree inferred from nucleotide sequences of 13 PCGs of the mitogenome using ML analysis [file zookeys-1240-257_article-141126__-s007.jpg]
